# *Hemipilia huanglongensis* (Orchidaceae), a new species from north Sichuan, China

**DOI:** 10.3897/phytokeys.273.181545

**Published:** 2026-04-22

**Authors:** Da-Jun Xie, Jin-Xiao Li, Tan Deng, Ruo-Xun Wei, Quan-Li Dou, Shu-Ming Peng, Ren-Bo Zhang

**Affiliations:** 1 Sichuan Academy of Forestry, Chengdu, 610081, China Zunyi Normal College Zunyi China; 2 Department of Biology, Zunyi Normal College, Zunyi, 563000, China Sichuan Academy of Forestry Chengdu China; 3 Ecological Environment College, Chengdu University of Technology, Chengdu, 610059, China Chengdu University of Technology Chengdu China

**Keywords:** Conservation assessment, morphological diagnosis, new species description, orchid systematics, Sichuan flora

## Abstract

A new species of Orchidaceae, *Hemipilia huanglongensis* D.J.Xie & R.B.Zhang, was discovered on steep karst slopes in northern Sichuan, China and is described and illustrated, based on morphological and molecular phylogenetic evidence. While morphologically similar to *H. amplexifolia* (Tang & F.T.Wang) Y.Tang and H.Peng, *H. faberi* (Rolfe) Y.Tang and H.Peng and *H. tetraloba* (Finet) Y.Tang and H.Peng, it can be distinguished by a squamose lip base with an upwardly protruding lamella, along with larger floral structures, including the sepals, petals, lip and ovary. Phylogenetic analyses, based on nuclear (ITS) and plastid (*mat*K, *rbc*L) DNA sequences, further support its distinction from these three morphologically allied species.

## Introduction

The genus *Hemipilia* Lindl. (Lindley 1835) *sensu stricto* is primarily distributed in the mountainous regions of south-western China and the Himalayas, extending southwards to Thailand, with nine species recorded in China (Lang 1999). Its range spans from Nepal through Bhutan and southern China to Myanmar and Thailand, including seven species (five endemic) within the country (Chen et al. 2009). *Hemipilia* s.s. is morphologically characterised by a protruding, tongue-like rostellum (Tang et al. 2016). However, molecular phylogenetic studies reveal that, although *Hemipilia* s.s. constitutes a monophyletic group, it is deeply embedded within a well-supported clade that also includes species historically placed in *Ponerorchis* (Reichenbach 1852) (with a key distinguishing feature being the presence of a bursicle enclosing the viscidium) and *Hemipiliopsis* (Luo and Chen 2003) (originally diagnosed by traits including column morphology and rostellar structure). This close phylogenetic relationship has prompted significant taxonomic re-consideration. While some studies have proposed treating *Ponerorchis* and *Hemipiliopsis* as synonyms within a broadly circumscribed *Hemipilia* (Tang et al. 2015), others have maintained them as distinct or merged them under different schemes (Jin et al. 2014; Jin et al. 2017).

In a comprehensive phylogenetic study of subtribe Orchidinae, Jin et al. (2014) proposed several generic re-circumscriptions, including the combination of *Hemipiliopsis* with *Hemipilia*. Within the same well-supported clade, their analysis also placed species of *Ponerorchis* as sister to *Hemipilia*, while *Amitostigma* (Schlechter 1919a) and *Neottianthe* (Schlechter 1919b) formed a separate subclade sister to the *Ponerorchis*-*Hemipilia* lineage. While recognising the close relationship, Jin et al. (2014) provisionally retained *Ponerorchis* as distinct from *Hemipilia*, citing the need for a more detailed morphological re-assessment and broader species sampling to confidently resolve their monophyly and relationships. Their primary argument for synonymising *Hemipiliopsis* with *Hemipilia* was the lack of strong molecular or consistent morphological support for its separation. Specifically, the diagnostic rostellar structure of *Hemipiliopsis* was found to be variable and not exclusive, falling within the morphological continuum of *Hemipilia* s.l. In contrast, the proposed combination of *Amitostigma* and *Neottianthe* with *Ponerorchis* was based on their nested phylogenetic position within *Ponerorchis* s.l. and shared key morphological traits, such as a short, saccate or spurless lip and a viscidium either naked or partially covered by a bursicle, which differed from the floral morphology typical of *Hemipilia*. For the scope of the present paper, which focuses on the taxonomy of *Hemipilia* and its immediate relatives, we follow the conclusions of Jin et al. (2014) and subsequent studies in treating *Hemipiliopsis* as a synonym of *Hemipilia*.

A subsequent comprehensive phylogenetic study by Tang et al. (2015) resolved the East Asia Clade (also referred to as the *Amitostigma* alliance) as a robust monophyletic group. They argued that this entire clade, which encompasses the traditional genera *Amitostigma*, *Hemipilia*, *Hemipiliopsis*, *Neottianthe*, *Ponerorchis* and *Tsaiorchis* (Tang and Wang 1936), amongst others, is best treated as a single genus. Applying nomenclatural priority, they adopted the oldest available name for this broadly defined genus, *Hemipilia* (termed “*Hemipilia sensu latissimo*” in their classification). This treatment contrasts with that of Jin et al. (2014), as Tang et al. (2015) considered the latter’s more narrowly defined *Ponerorchis* s.l. (comprising only their Clades N5–N7) to be weakly supported and morphologically heterogeneous.

Subsequent to the broader treatment of Tang et al. (2015), who advocated for a single genus *Hemipilia* to encompass the entire *Amitostigma* alliance, Jin et al. (2017) proposed an alternative, more conservative classification for this group (which they termed the *Ponerorchis* alliance). They recognised five distinct genera: *Hemipilia* (in a narrower sense), *Sirindhornia* (Pedersen and Suksathan 2002), *Shizhenia* (Jin et al. 2015), *Tsaiorchis* and a broadly defined *Ponerorchis* s.l. This *Ponerorchis* s.l. explicitly included species previously classified under *Amitostigma* and *Neottianthe*, based on their earlier phylogenetic work (Jin et al. 2014). The decision by Jin et al. (2017) to not adopt the single-genus system of Tang et al. (2015) reflected a different taxonomic philosophy, prioritising morphologically diagnosable and comparatively narrower generic limits.

In this study, we follow a broad circumscription of *Hemipilia* that aligns with the monophyletic concept of Tang et al. (2015), but is refined, based on subsequent taxonomic updates and nomenclatural stability. Our treatment recognises *Hemipilia* as encompassing the former genera *Amitostigma*, *Hemipiliopsis*, *Neottianthe*, *Ponerorchis* and *Shizhenia*, which is consistent with the current delineation in the International Plant Names Index (IPNI 2026). This framework provides a stable basis for the taxonomic discussion and new combinations proposed in this work.

In July 2025, a plant, morphologically similar to *H. amplexifolia* (Tang & F.T.Wang) Y.Tang and H.Peng, *H. faberi* (Rolfe) Y.Tang and H.Peng and *H. tetraloba* (Finet) Y.Tang and H.Peng, was discovered in Sichuan Province, China. Through careful examination of flowering specimens in the laboratory and detailed observation of living plants, significant morphological distinctions between this plant and the three known species were identified. A review of the relevant literature led to its treatment as a new species within the genus *Hemipilia*. Phylogenetic analyses, based on internal transcribed spacer (ITS), plastid maturase K (*mat*K) and RuBisCO large subunit (*rbc*L) sequences, further confirmed that it is distinct from its morphologically closest relatives.

## Material and methods

### Taxonomic revision

Specimens for this study were collected at Huanglong Natural Reserve, Songpan City (Sichuan, China), the type locality. Voucher specimens have been deposited in the Botany Herbarium at Zunyi Normal College (ZY) and the Herbarium of the Sichuan Academy of Forestry (**SCFI**) (codes referring to Thiers (2026)). Using a stereomicroscope (Olympus Optical Microscope SZ61, Olympus Corp., Tokyo, Japan), we conducted micromorphological analyses and photography. We compared the morphological traits with the protologue and type specimens of previously described *Hemipilia* species, particularly new *Hemipilia* taxa from Sichuan and nearby provinces, along with herbarium specimens at relevant herbaria (Suppl. material 1). High resolution *Hemipilia* specimens were examined from IBK, IBSC and PE in the plant sub-platform of the National Specimen Information Infrastructure platform (Ministry of Science and Technology of China 2013). We also studied colour photographs of *Hemipilia* presented on the Plant Photo Bank of China website (Institute of Botany of Chinese Academy of Sciences 2008).

### Phylogenetic analysis

Leaf material of the undescribed species was collected in Huanglong Natural Reserve, Songpan City (Sichuan, China) and promptly silica-dried for DNA extraction. The nuclear ribosomal ITS region, as well as the *mat*K and *rbc*L regions, were utilised in this study. Following Lin et al. (2021), we conducted DNA extraction, employed primers and conducted PCR amplification and sequencing. The data reported in this paper have been deposited in the GenBase (Bu et al. 2024) in the National Genomics Data Center (Members and Partners 2024), Beijing Institute of Genomics, Chinese Academy of Sciences/China National Center for Bioinformation. The associated accession numbers for the taxonomic novelty are publicly accessible at https://ngdc.cncb.ac.cn/genbase as follows: ITS, C_AA132255; *mat*K, C_AAM15018; *rbc*L, C_AA131592.

To elucidate the phylogenetic affinities of the genus, we integrated 41 *Hemipilia* species (Table 1). Based on previous phylogenetic analyses (Lin et al. 2021), two *Habenaria* Willd. (Willdenow 1805) species (*H. petelotii* Gagnep. and *H. aitchisonii* Rchb.f.) were selected as outgroups and two non-*Hemipilia* species (*Sirindhornia pulchella* H.A.Pedersen & Indham. and *Tsaiorchis keiskeoides* (Gagnep.) X.H.Jin, Schuit. & W.T.Jin) were selected as ingroups. The relevant gene sequences were sourced from the National Center for Biotechnology Information (https://www.ncbi.nlm.nih.gov/) (except for those of the novel species, which are deposited in GenBase).

**Table 1.** Accession numbers from GenBank and GenBase used in this study. Newly-reported sequences are highlighted in bold, while a dash (“–”) is used to represent missing data.

Species	ITS	*mat*K	*rbc*L
*Habenaria aitchisonii*	MW775135	MW924318	-
*Habenaria petelotii*	MF944311	MF945526	MF944962
*Hemipilia alpestris*	KJ460093	KJ452849	KJ451547
*Hemipilia amplexifolia*	KM651222	KM651386	-
*Hemipilia basifoliata*	MF944399	MF945455	MF944889
*Hemipilia camptoceras*	MF944400	MF945409	MF944845
*Hemipilia capitata*	KM651224	KM651388	-
*Hemipilia chidori*	KM651287	KM651451	-
*Hemipilia chusua*	KJ460034	KJ452786	KJ451484
*Hemipilia compacta*	JN696455	KJ452796	KJ451494
*Hemipilia cucullata*	JN696456	KJ452792	KJ451490
*Hemipilia faberi*	KM651229	KM651389	-
*Hemipilia farreri*	KJ460047	KJ452803	KJ451501
*Hemipilia gongshanensis*	MW039579	MW045192	MW045193
*Hemipilia gracilis*	KM651236	KF262014	-
*Hemipilia hemipilioides*	KM651238	KM651400	-
***Hemipilia huanglongensis***	**C_AA132255**	**C_AAM15018**	**C_AA131592**
*Hemipilia jooiokiana*	KT338779	KF695169	-
*Hemipilia keiskei*	KM651239	KM651401	-
*Hemipilia kinoshitae*	KM651241	KM651403	-
*Hemipilia kiraishiensis*	MF944403	MF945445	MF944879
*Hemipilia lepida*	KM651242	KM651404	-
*Hemipilia limprichtii*	KM651297	MF945425	MF944861
*Hemipilia monantha*	KM651244	KM651407	-
*Hemipilia monophylla*	JN696454	KJ452791	KJ451489
*Hemipilia nana*	MF944404	MF945475	MF944908
*Hemipilia oblonga*	MF944405	MF945472	MF944906
*Hemipilia omeishanica*	KM651299	KM651464	-
*Hemipilia papilionacea*	KM651246	KM651408	-
*Hemipilia parceflora*	KJ460052	KJ452808	KJ451506
*Hemipilia physoceras*	KM651248	KM651410	-
*Hemipilia pinguicula*	MF944417	MF945495	MF944931
*Hemipilia secundiflora*	MF944406	MF945458	MF944892
*Hemipilia sichuanica*	KJ460059	KJ452815	KJ451513
*Hemipilia simplex*	MF944407	MF945427	MF944863
*Hemipilia suzukiana*	KM651300	KM651459	-
*Hemipilia tetraloba*	MF944411	MF945440	MF944875
*Hemipilia thailandica*	KM651256	KM651419	-
*Hemipilia tibetica*	MF944413	MF945490	MF944925
*Hemipilia trifurcata*	KJ460055	KJ452811	KJ451509
*Hemipilia wenshanensis*	KM651258	KM651422	-
*Hemipilia wolongensis*	MZ098270	-	-
*Hemipilia yuana*	KM651259	KJ452848	KJ451546
*Sirindhornia pulchella*	KJ460045	KJ452801	KJ451499
*Tsaiorchis keiskeoides*	KM651240	KM651402	-

The conflict between nrDNA and plastid DNA data was assessed in PAUP (https://paup.phylosolutions.com/) using the length difference test (LDT). Due to the significant incongruence detected between the two datasets, phylogenetic trees were constructed separately, based on nrDNA and plastid DNA markers, respectively. The aligned matrix comprised 646 base pairs for ITS and 2398 base pairs for the combined plastid markers (*mat*K and *rbc*L). Sequence alignment, model selection, super matrix construction, model selection and Bayesian Inference (BI) were performed using Phylosuite v.1.2.2 (Zhang et al. 2020). The best-fit molecular evolution models were selected under the Corrected Akaike Information Criterion (AICc) (GTR+F+I+G4 for the ITS and GTR+F+I for the two plastid markers). The BI analyses entailed four Markov Chain Monte Carlo chains, with tree sampling every 1000 generations for 1,000,000 generations from a random tree. Upon stabilising the log-likelihood scores, a consensus tree was computed, excluding 5,000 sampled trees as burn-in (Xie et al. 2014). The tree was visualised using tvBOT v. 2.6.1 (Xie et al. 2023).

The relevant data have been submitted and are publicly available on TreeBase at the following URL: http://purl.org/phylo/treebase/phylows/study/TB2:S32491.

## Taxonomic treatment

### Hemipilia huanglongensis

Taxon classification

Plantae

AsparagalesOrchidaceae

D.J.Xie & R.B.Zhang sp. nov.

71E990EB-91AA-5370-AA2A-FB60D17DFCC0

urn:lsid:ipni.org:names:77378951-1

Figs 1, 2

#### Type.

China • Sichuan Province, Songpan City, Huanglong Nature Reserve, elev. ca. 3500 m, 32.81722, 103.90306, growing on sunny, rocky slopes, 25 July 2025; *Ren-Bo Zhang ZRB3137* (fl.) (**holotype**: ZY!) and *Da-Jun Xie SCHL05* (fl.) (**paratypes**: SCFI!).

**Figure 1.**
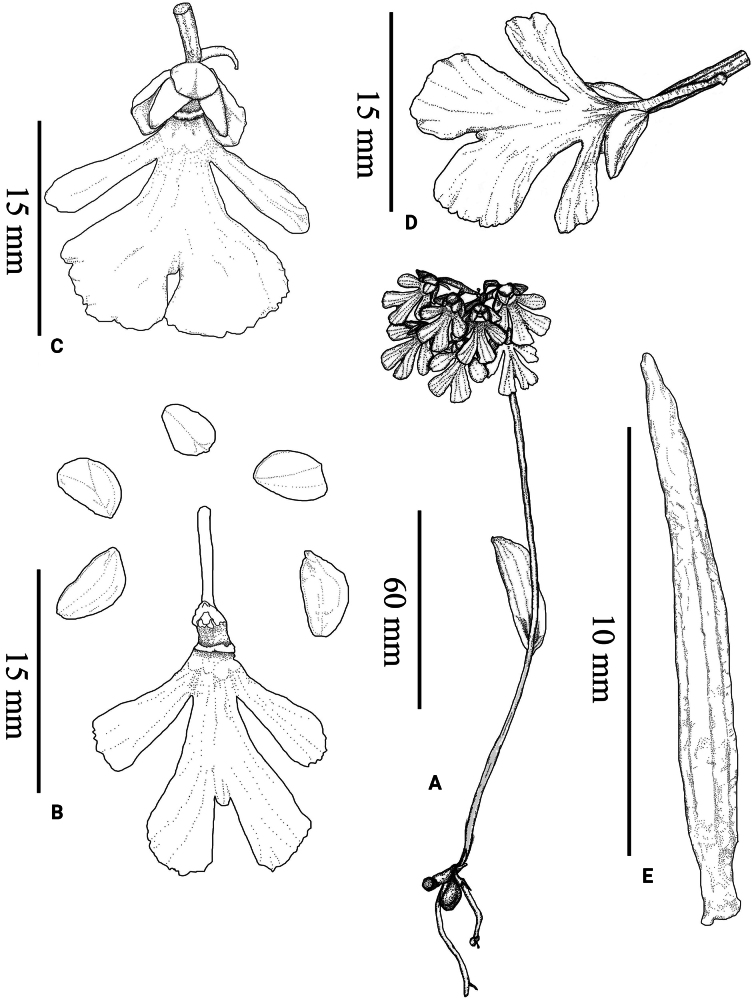
Line drawing of *Hemipilia huanglongensis* D.J.Xie & R.B.Zhang, sp. nov. **A**. Flowering plant; **B**. Floral dissection; **C**. Front flower view; **D**. Back flower view; **E**. Ovary and pedicel. Drawings from the type specimens by Tan Deng.

**Figure 2.**
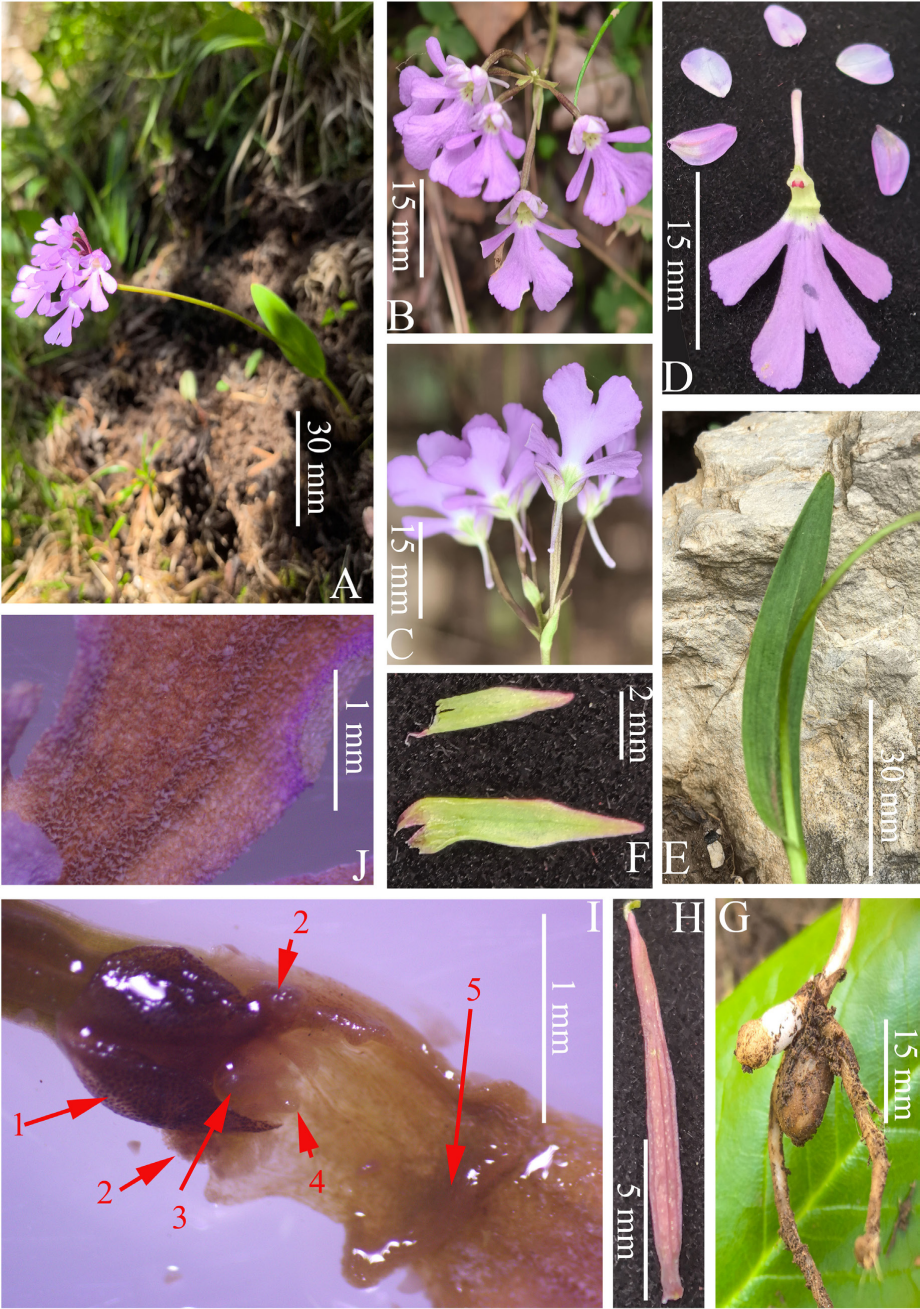
Images of living, dry or rehydrated specimens of *Hemipilia huanglongensis* D.J.Xie & R.B.Zhang. **A**. Flowering plant; **B**. Front inflorescence view; **C**. Back inflorescence view; **D**. Flower disintegration; **E**. Leaf; **F**. Bracts; **G**. Tubers; **H**. Ovary and pedicel; **I**. Gynostemium (1: anther; 2: auricle; 3: rostellum; 4: bursicle; 5: lamellae at lip base); **J**. Squamose lip base (Photographed by T. Deng and R.B. Zhang).

#### Diagnosis.

*Hemipilia huanglongensis* morphologically resembles *H. amplexifolia*, *H. faberi* and *H. tetraloba*. *Hemipilia huanglongensis* differs from *H. amplexifolia* in having a multi-flowered rachis (vs. 1–2-flowered) and purple flowers (vs. white with purplish-red spotting, which may appear red in PPBC photographs). *Hemipilia huanglongensis* can be distinguished from the other two species by the lip base (squamose vs. papillate in *H. faberi* and *H. tetraloba*, following the same order) with an upwardly protruding lamellae (vs. absent), more lateral sepal veins (3 vs. 1), longer ovary and pedicel (≥ 12.6 vs. ≤ 10; measurements in millimetres, the same below), longer dorsal sepal (≥ 3.7 vs. ≤ 3.5), longer lateral sepal (≥ 4.7 vs. ≤ 4), longer petal (≥ 3.7 vs. ≤ 3.5), longer lip (≥ 12.6 vs. ≤ 8), wider lip (≥ 8.3 vs. ≤ 7.5), longer lateral lip lobe (≥ 4.5 vs. ≤ 3.8) and longer middle lip lobe (≥ 8.6 vs. ≤ 4.2).

#### Type.

***Herbs***, 7–28 cm tall. ***Tubers*** ovate-elliptical, 6–10 mm long. ***Stems*** with 1 or 2 tubular sheaths at base, 1-leafed. ***Leaves*** cauline, porrect, narrowly lanceolate to elliptic or ovate, 3–8 × 0.5–0.8 cm, apex obtuse. ***Peduncle*** ebracteate; rachis 6.5–13 cm long, several flowered; floral bracts lanceolate, 4.6–7.7 mm long, much shorter than ovary, apex acuminate. ***Flowers*** often secund, purple; ovary and pedicel 12.6–20.0 mm long. ***Dorsal sepal*** oblong-ovate, 3.7–4.0 × 2.4–2.6 mm, 1-veined, apex obtuse; ***lateral sepals*** oblique ovate, 4.7–5.8 × 2.5–3.0 mm, 3-veined, margin somewhat undulate, apex obtuse. ***Petals*** loosely connivent with dorsal sepal and forming a hood, sub-orbicular, concave, slightly oblique, 3.7–4.6 × 3.0–3.6 mm, 1-veined with several shorter veins; ***lip*** broadly ovate, 12.6–15.3 × 8.3–12.5 mm, base cuneate and with an upwardly protruding lamellae, 3-lobed below middle; disc densely papillate; ***lateral lobes*** oblanceolate, 4.5–6.0 × 1.6–3.0 mm, apical margin irregularly crenate, apex obtuse; ***mid-lobe*** obovate, 8.6–11.0 × 5.8–9.0 mm, base cuneate, apical margin irregularly crenate, deeply notched towards apex to form 2 distinct lobules, sinus with an obtuse tooth; ***spur*** nearly horizontal, cylindrical-clavate, 5.1–8.0 mm long, slightly dilated towards apex, apex obuse; ***viscidium*** oblong; stigma lobes clavate.

#### Phenology.

Flowering occurs in July.

#### Etymology.

The specific epithet is derived from the type locality, Huanglong Nature Reserve, Sichuan Province, China.

#### Vernacular name.

The Chinese name proposed here is “黄龙舌喙兰”. Phonetically, it is “huáng lóng shé huì lán”.

#### Distribution and ecology.

The new species is currently known only from the type locality, the Huanglong Nature Reserve in Songpan County, Aba Prefecture, Sichuan Province. It grows on rocky, sun-exposed slopes, at an altitude of ca. 3500 m.

#### Conservation status.

*Hemipilia huanglongensis* is known only from the type locality, with the individuals estimated to be more than several hundreds of plants. The known subpopulations are restricted to a very small area within the Huanglong Valley, resulting in a very limited extent of occurrence (EOO). The estimated area of occupancy (AOO) is also restricted and the total number of mature individuals is low. Although the habitat is currently within a protected area and not under immediate anthropogenic threat, the species meets the criteria for Vulnerable (VU) under criterion D2 (population with a very restricted area of occupancy or number of locations, making it prone to the effects of human activities or stochastic events within a very short time period) (IUCN Standards and Petitions Committee 2022). A provisional conservation status of VU D2 is therefore proposed.

#### Taxonomic and phylogenetic notes.

**Figure 3.**
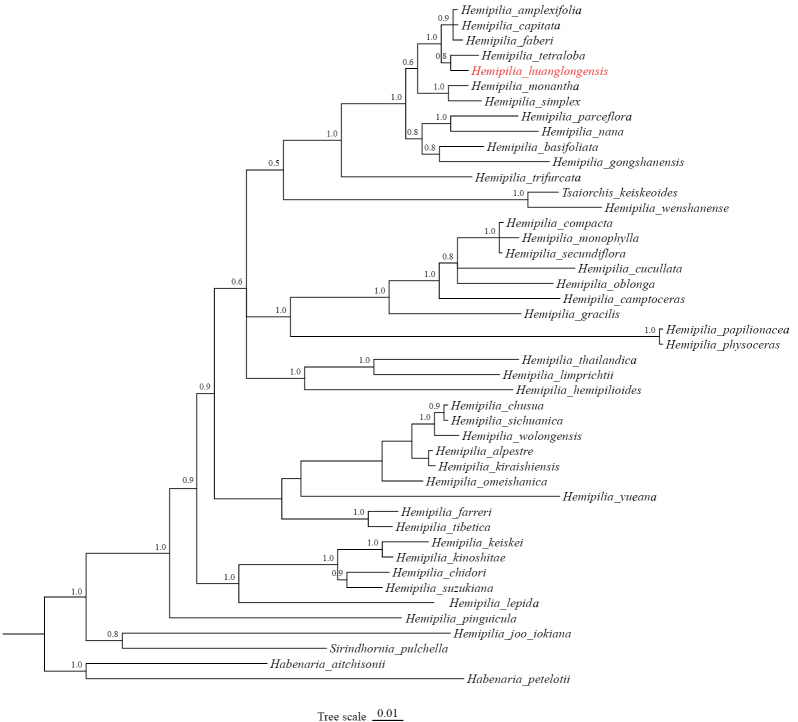
The Bayesian phylogenetic tree of *Hemipilia*, including *H. huanglongensis*, is based on nrITS sequence data, with branch lengths reflecting nucleotide substitution rates. Posterior probabilities (PP) are indicated alongside branches and the new species is highlighted in red.

**Figure 4.**
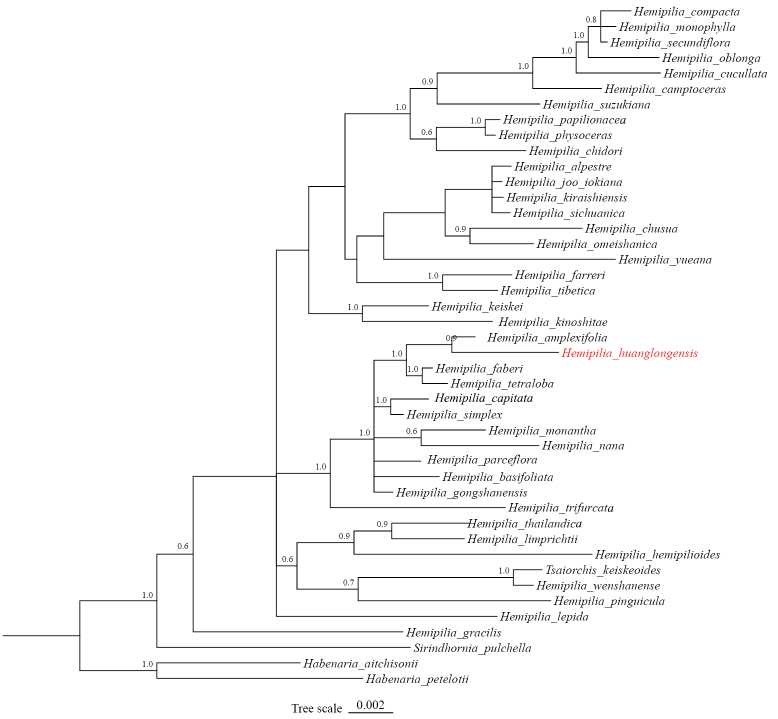
The Bayesian phylogenetic tree for *Hemipilia*, incorporating *H. huanglongensis*, was reconstructed using combined *matK* and *rbcL* plastid data, with branch lengths scaled to nucleotide substitution rates. Posterior probabilities (PP) are shown adjacent to branches and the newly-described species is marked in red.

*Hemipilia huanglongensis* is close to *H. amplexifolia*, *H. faberi* and *H. tetraloba*, based on morphological characters, yet it is clearly differentiated from them by the characters listed in Table 2. Although conflicts exist between the ITS and plastid data, the clade comprising *H. huanglongensis* together with *H. amplexifolia*, *H. faberi*, and *H. tetraloba* is consistently recovered in both the nuclear and plastid trees. This result is congruent with previous studies (Jin et al. 2014; Tang et al. 2015), providing further support for the close relationship amongst these species. The BI of ITS showed that *H. huanglongensis* was nested within the *H. tetraloba*-*H. amplexifolia* clade with high support (Fig. 3, PP = 1.0). Similarly, analysis of its plastid counterpart indicated that *H. huanglongensis* is close to *H. amplexifolia* with high support (Fig. 4, PP = 0.9), whose clade was sister to a clade formed by *H. faberi* and *H. tetraloba* with high support (Fig. 4, PP = 1.0).

**Table 2.** Morphological comparison of *Hemipilia huanglongensis*, *H. amplexifolia*, *H. faberi* and *H. tetraloba*.

Characters	*Hemipilia huanglongensis*	*H. amplexifolia*	*H. faberi*	*H. tetraloba*
Peduncle bract	0	(Not described)	1	0 or 1
Flower count per rachis	Several	1–2	Several to more than 10-flowered	3- to more than 10-flowered
Flower colour	Purple	White with purplish-red spots (also observed as red in the PPBC)	Pinkish-purple	Pink to pale purple
Ovary and pedicel length (mm)	12.6–20.0	8–10	8–10	6–9
Dorsal sepal length (mm)	3.7–4.0	Ca. 3.5	2.5–3.5	2.5–3.3
Lateral sepal length (mm)	4.7–5.8	Ca. 4	3–4	3–4
Lateral sepal veins	3	(Not described)	1	1
Petal length (mm)	3.7–4.6	Ca. 3	2–3	2.8–3.5
Lamellae at lip base	**Present**	Absent	Absent	Absent
Appendage at lip base	Squamose	(Not described)	Papillate	Papillate
Lip length (mm)	12.6–15.3	5–6	6–7.5	4.5–6(–8)
Lip width (mm)	8.3–12.5	4–5	6.2–7.5	3.5–4
Later lip lobe length (mm)	4.5–6.0	(Not described)	2.5–3.8	2–3.5
Middle lip lobe notch	Deep	Deep	Deep	Shallow or truncate
Middle lip lobe length (mm)	8.6–11.0	(Not described)	3–4.2	2.5–4

## Supplementary Material

XML Treatment for Hemipilia huanglongensis

## Additional information

### Use of AI

No use of AI was reported.

### Author ORCIDs

Da-Jun Xie https://orcid.org/0009-0008-4030-4718

Jin-Xiao Li https://orcid.org/0009-0000-7600-3329

Tan Deng https://orcid.org/0000-0002-4575-3298

Ruo-Xun Wei https://orcid.org/0009-0000-0510-7470

Quan-Li Dou https://orcid.org/0009-0003-8462-7774

Shu-Ming Peng https://orcid.org/0009-0009-5187-4676

Ren-Bo Zhang https://orcid.org/0000-0002-9860-5803

### Data availability

All of the data that support the findings of this study are available in the main text or Supplementary Information.

## Supplementary materials

Supplementary material 1 Compared the morphological traits with the protologue and type specimens of previously described *Hemipilia* species Data type pdf Explanation note Compared the morphological traits with the protologue and type specimens of previously described *Hemipilia* species, particularly new *Hemipilia* taxa from Sichuan and nearby provinces, along with herbarium specimens at relevant herbaria. https://binary.pensoft.net/file/1602868This dataset is made available under the Open Database License (http://opendatacommons.org/licenses/odbl/1.0/). The Open Database License (ODbL) is a license agreement intended to allow users to freely share, modify, and use this Dataset while maintaining this same freedom for others, provided that the original source and author(s) are credited. Da-Jun Xie, Jin-Xiao Li, Tan Deng, Ruo-Xun Wei, Quan-Li Dou, Shu-Ming Peng, Ren-Bo Zhang
