# Supplementary material for: Hemipilia huanglongensis (Orchidaceae), a new species from north Sichuan, China
Source: PhytoKeys. 2026 Apr 22;273:225–36. doi: 10.3897/phytokeys.273.181545 (PMC13129549; doi:10.3897/phytokeys.273.181545)
Supplement: Supplementary material 1 — Compared the morphological traits with the protologue and type specimens of previously described Hemipilia species [file phytokeys-273-225_article-181545__-s001.pdf]

Taxon	Herbarium Code	Collector	Collection No.	Barcode	Date	Location
Amitostigma amplexifolium	PE	Wilson	4574	00200209	19030700	China
Amitostigma faberi	IBK	熊济华，张秀实，蒋兴麐	31512	IBK00144609	19520000	Sichuan, China
Amitostigma faberi	IBK	方文培	31751	IBK00144610	19520000	Sichuan, China
Amitostigma faberi	IBSC	T.T.Yu	1299	0623532	19330000	Sichuan, China
Amitostigma faberi	IBSC	F.T.Wang	23408	0623531	19310722	Yunnan, China
Amitostigma faberi	IBSC	熊济华，张秀实，蒋兴麐	31751	0623530	19520723	Emei County, Sichuan, China
Amitostigma faberi	KUN	张挺，郭永杰，张玉武等	10CS1747	1396693	20100613	Jiangkou County, Guizhou, China
Amitostigma faberi	NAS	W.P.Fang	4722	NAS00556806	N	China
Amitostigma faberi	PE	郎楷永，高宝莼等	8	00200228	19800701	Emei City, Sichuan, China
Amitostigma faberi	PE	王中仁	854	00200227	19710803	Emei City, Sichuan, China
Amitostigma faberi	PE	四川任务组	2038	00200223	19710802	Emei City, Sichuan, China
Amitostigma faberi	PE	姚仲吾	2328	00200233	19380116	Hongya County, Sichuan, China
Amitostigma faberi	PE	汪发缵	23408	00200229	19310722	Emei City, Sichuan, China
Amitostigma faberi	PE	唐进	23408	00200231	19310722	Emei City, Sichuan, China
Amitostigma faberi	PE	熊济华，蒋兴麟，张秀实	31751	00200234	19520723	Emei City, Sichuan, China
Amitostigma faberi	PE	宋滋圃	38617	00200232	19540000	Baoxing County, Sichuan, China
Amitostigma tetralobum	IBSC	杨光辉	58767	0623528	19580714	Wuxi County, Sichuan, China
Amitostigma tetralobum	PE	王中仁，马义伦，李景成	392	00200362	19840612	Dali City, Yunnan, China
Amitostigma tetralobum	PE	钟观光	2293	00200356	19080903	Dali City, Yunnan, China
Amitostigma tetralobum	PE	王汉臣	2397	00200360	19420619	Yangbi County, Yunnan, China
Amitostigma tetralobum	PE	俞德浚	7677	00200350	19370812	Muli County, Sichuan, China
Amitostigma tetralobum	PE	俞德浚	7677	00200351	19370812	Muli County, Sichuan, China
Amitostigma tetralobum	PE	俞德浚	7677	00200352	19370812	Muli County, Sichuan, China
Amitostigma tetralobum	PE	G. Forrest	11724	00200355	19130000	Dali City, Yunnan, China
Amitostigma tetralobum	PE	Anonymous	13084	00200353	19760722	Meigu County, Sichuan, China
Amitostigma tetralobum	PE	王启无	63409	00200359	19350500	Dali City, Yunnan, China
Amitostigma tetralobum	PE	王汉臣	2397B	00200361	19420619	Yangbi County, Yunnan, China
Amitostigma tetralobum	PE	Anonymous	s.n.	00200354	N	Sichuan, China
